# Composition, Antifungal, Phytotoxic, and Insecticidal Activities of *Thymus kotschyanus* Essential Oil

**DOI:** 10.3390/molecules25051152

**Published:** 2020-03-04

**Authors:** Ghader Ghasemi, Abolfazl Alirezalu, Youbert Ghosta, Azadeh Jarrahi, Seyed Ali Safavi, Mahdi Abbas-Mohammadi, Francisco J. Barba, Paulo E. S. Munekata, Rubén Domínguez, José M. Lorenzo

**Affiliations:** 1 Department of Horticultural Sciences, Faculty of Agriculture, Urmia University, Urmia 5756151818, Iran; gader.g1390@yahoo.com; 2 Department of Plant Protection, Faculty of Agriculture, Urmia University, Urmia 165-5715944931, Iran; ghoosta@gmail.com (Y.G.); jarrahiazadeh@yahoo.com (A.J.); a.safavi@urmia.ac.ir (S.A.S.); 3 Department of Phytochemistry, Medicinal Plants and Drugs Research Institute, Shahid Beheshti University, Tehran 1983969411, Iran; mehdiamohamadi@ymail.com; 4 Nutrition and Food Science Area, Preventive Medicine and Public Health, Food Sciences, Toxicology and Forensic Medicine Department, Faculty of Pharmacy, Universitat de València, Avda. Vicent Andrés Estellés, s/n, 46100 Burjassot, València, Spain; francisco.barba@uv.es; 5 Centro Tecnológico de la Carne de Galicia, rúa Galicia n° 4, Parque Tecnológico de Galicia, 16 San Cibrao das Viñas, 32900 Ourense, Spainrubendominguez@ceteca.net (R.D.)

**Keywords:** monoterpenes, thymol, γ-terpene, crop pests, post-harvest management

## Abstract

Essential oils (EOs) are some of the outstanding compounds found in *Thymus* that can exert antifungal, phytotoxic, and insecticidal activities, which encourage their exploration and potential use for agricultural and food purposes. The essential oils (EO) obtained from *Thymus kotschyanus* collected in the East Azerbaijan Province (Iran) were characterized using a gas chromatography-mass spectrometry (GC-MS) analysis. Thymol was the most important compound (60.48%), although 35 other active compounds were identified in the EO. Significant amounts of carvacrol (3.08%), *p*-cymene (5.56%), and γ-terpinene (6.67%) were found in the EO. The *T. kotschyanus* EO was tested against important phytopathogenic fungi (*Botrytis cinerea, Aspergillus niger*, and *Penicillium expansum*). The antifungal assay showed that the use of ≥500 ppm of EO resulted in a fungicidal effect against all funguses tested. In a similar way, the use of ≥500 ppm of EO inhibited the germination of all crop weed seeds (*Amaranthus retroflexus* L. and *Panicum miliaceum* L.) and their subsequent growth, which demonstrated its herbicidal effect. Finally, the insecticidal capacity of *T. kotschyanus* EO was also observed against selected insects (*Oryzaephilus surinamensis* and *Sitophilus oryzae*). *O. surinamensis* was more susceptible to the effect of EO (LC_50_ = 4.78 µL/L air) than *S. oryzae* (LC_50_ = 13.20 µL/L air). The obtained results of the present study can provide new safe resources to the development of new products for the food, agriculture, and pharmaceutical industries.

## 1. Introduction

The Genus *Thymus* L., family Labiatae, consists of more than 215 herbaceous perennial species mainly distributed in the Mediterranean region [1,2]. In Iran, fourteen species of *Thymus* L. are known [3]. *Thymus kotschyanus* represents one of the most diffused *Thymus* species. It is exploited mainly, but not only, in folk medicine to improve the digestive process and to treat respiratory disorders. Furthermore, its use as an aromatic ingredient, spice, or to prepare an herbal tea is well-known [2]. Aromatic and medicinal plants have a variety of biological compounds, including essential oils, alkaloids, coumarins, flavonoids, phenols, saponins, and tannins, which provide them with antibacterial, antifungal, and pesticide attributes [4]. Essential oils (EOs) represent a sub-category of secondary metabolites which are found in aromatic plants, known as natural and various compounds with significant attributes and which play a key role in protecting the plants via their antibacterial, antiviral, antifungal, and pesticide attributes [5]. These EOs are a complex mixture of substances that are present at different forms and concentrations. Monoterpenes, oxygenated monoterpenes, sesquiterpenes, and oxygenated sesquiterpenes are the predominant constituents, but phenolic compounds are also important components of some EOs [6]. The EO’s chemical composition is varied, because it is affected by environmental conditions, ontogenetic, weather and temperature, pre and post-harvest, and genetic factors [7]. With regards to this, there are a significant variation in chemical compositions of the EOs of various *Thymus* species. A recent research found that the essential oil composition of *T. kotschyanus* contained 54.6% of thymol [8], while other authors found that the EOs of this species was composed by 41.4% of carvacrol and 19.6% of thymol [9]. Other *Thymus* species, such as *T. carnosus*, *T. pubescens*, and *T. persicus*, showed values of thymol between 14% and 36% [10,11,12]. In addition to thymol, in some of these species, authors also observed high contents of other compounds, such as *p*-cymene (21.3%) [12], carvacrol (48.8%) [11], and geraniol (9.4%) [10].

On the other hand, several EOs and other plant extracts are also used as substitutes for synthetic additives in the food industry [13,14,15], by direct addition [16,17,18,19,20] or by applying as active packaging [21,22,23], to limit microbial and oxidation degradation of food [24,25,26,27].

Over the last decade, *T. kotschyanus* has gained popularity due to its potential applications, not only in the food industry but also in the pharmaceutical (ascertained antifungal, anti-inflammatory, antimicrobial, and expectorant properties) and cosmetic industries, as components of soaps, toothpastes, and perfumes [1,28,29,30]. In addition, the management of crop pests and diseases is a constant and necessary concern in agricultural and food industries. For instance, *Botrytis cinerea*, *Penicillium expansum*, and *Aspergillus niger* are among the most common worldwide post-harvest pathogens of fruits and vegetables [31].

However, synthetic pesticides, herbicides, and fungicides are contaminants introduced into the environment that can be extremely hazardous to the human body and agricultural products due to slow degradation and consequent persistence in the environment [32,33]. Moreover, excessive use of synthetic pesticides, herbicides, and fungicides has made pathogens and insects to be resistant to such chemicals [34,35]. In this line of thought, the search for environmentally friendly strategies to manage crop pests and diseases has become an interesting field of application for natural products [32,33]. Among the green strategies, the use of EOs is an appealing alternative to synthetic and toxic compounds commonly used in crop management and, for instance, as new antifungal agents [36]. Since some EOs from medicinal herbs exhibit phytotoxic activity, these natural sources can be explored for agricultural and food-processing purposes [4,37].

Essential oils (EOs) are some of the outstanding compounds found in *Thymus* L. that can exert antifungal, bactericidal, phytotoxic, antiparasitic, and insecticidal activities, which encourage their exploration and potential use for agricultural and food purposes [4,16,37,38,39]. In the last decades, EOs were also studied to evaluate their use in weed control and crop productivity (the so-called allelopathic effect) [40]. The compounds with such properties could be used for the biological control of plant pathogens and weeds as natural pesticides and herbicides with less destructive effects on the environment [41]. Different EOs obtained from some thyme species have demonstrated allelopathic effects and could be used to control and combat the growth of the weed species [42,43].

Although several *Thymus* species were studied and the chemical composition, antioxidant activity, and biological properties of their essential oils were determined [29,44], few studies deal with the composition and biological activity of *T. kotschyanus* in North West Iran. Therefore, in the present study, the antioxidant, phytochemical, antifungal, phytotoxic, and insecticidal properties of some derivatives of *T. kotschyanus* grown in the North West of Iran were evaluated.

## 2. Results and Discussion

### 2.1. Phytochemical Characterization

**Figure 1:**
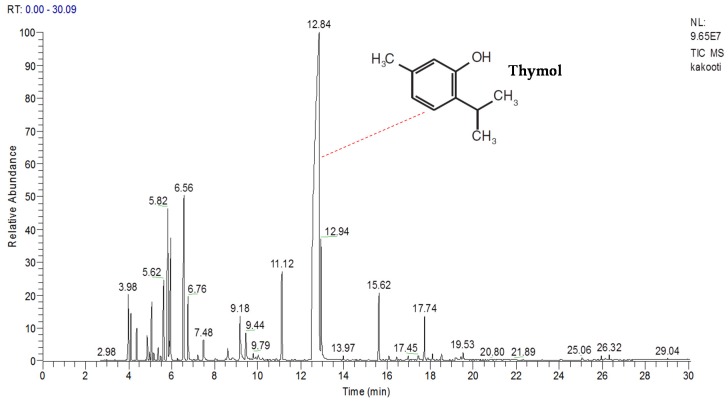
Gas chromatography-mass spectrometry chromatograms of *Thymus kotschyanus* essential oil (EO) from Shabestar, East Azerbaijan, Iran.

The amount of essential oil (EO) yielded by the Clevenger hydro-distillation method was 3.5% (*v*/*w*) of dry weight, which had a color nuance ranging from light orange to yellow. GC-MS chromatogram and chemical compositions of EO are shown in Table 1 and Figure 1, respectively.

A total of 36 compounds were detected by GC-MS analysis, being thymol (60.48%), γ-terpinene (6.67%), *p*-cymene (5.56%), and carvacrol (3.02%), as well as 1,8-cineol (2.82%) and E-caryophyllene (2.18%) as the main components. The extraction yield of *T. kotschyanus* EO is in the range of values reported for Thyme species, which can vary in the range of 0.8–2.6% [45,46,47,48,49,50]. Moreover, among 14 *Thymus* samples from Iran studied by Tohidi et al. [51], EO yield varied from 0.29% (*T. fedtschenkoi*) to 3.87% (*T. migricus*).

The yield of EO and thymol content found in this study were significantly higher compared to other previous studies evaluating *T. kotschyanus*. Therefore, *T. kotschyanus* collected from the Shabestar region could be identified as a new thymol chemotype. For instance, other authors found variable amounts in *T. kotschyanus* that ranges from 1.1% in plants collected in Yarz (Iran) [52] to 54.66% in plants collected in the highlands of Bojnurd (Iran) at an altitude of 1700 m [8]. In addition, Tohidi et al. [53] analyzed the EOs of ten *Thymus* species from different areas of Iran and reported that thymol contents ranged from 12.4% (EO of *T. fedtschenkoi*) to 79.74% (EO of *T. migricus*).

### 2.2. Antifungal Activity

**Figure 2:**
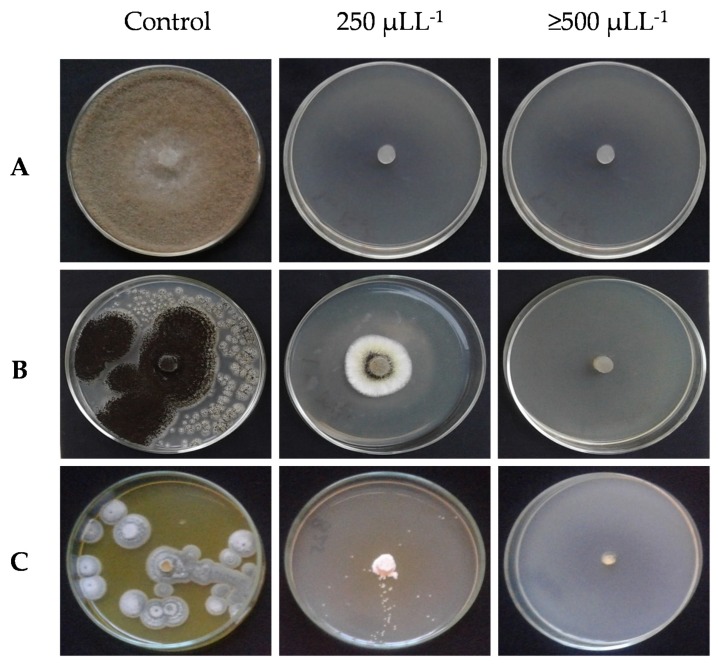
Antifungal activity of different concentration of *T. kotschyanus* EO on *Botrytis cinerea* (**A**), *Penicillium expansum* (**B**), and *Aspergillus niger* (**C**).

**Figure 3:**
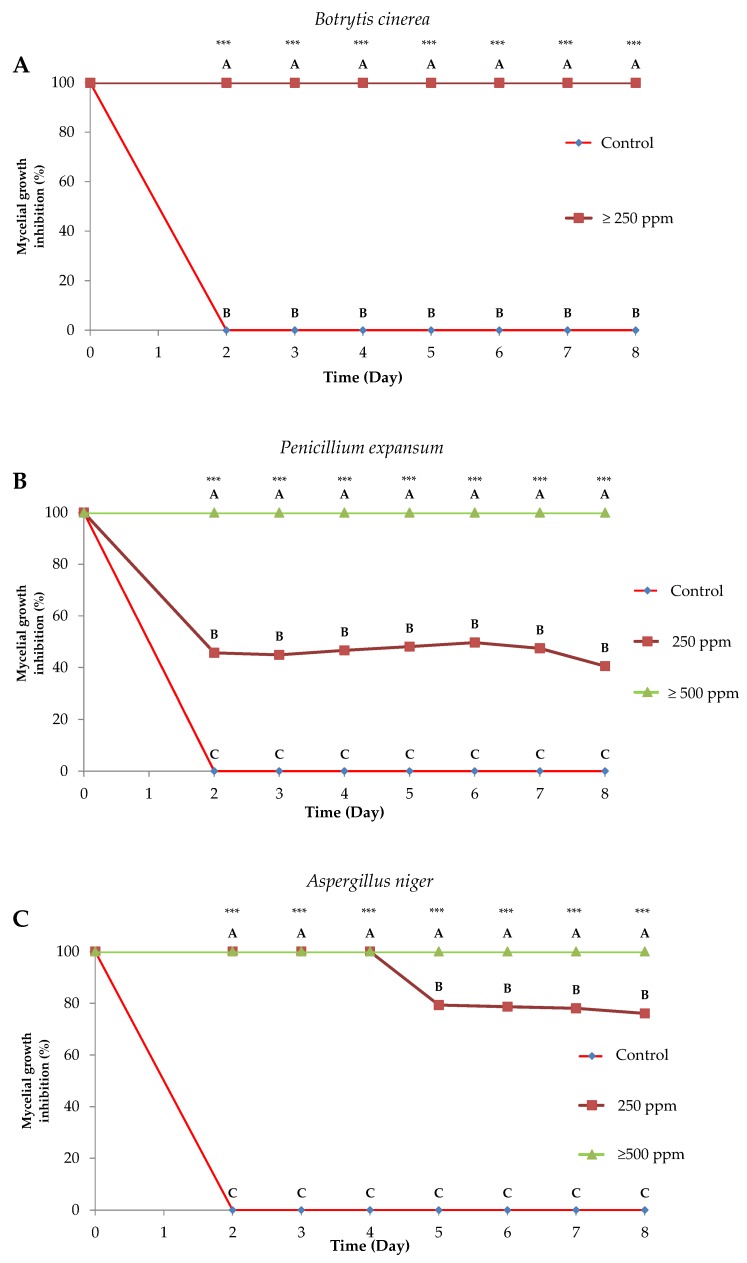
Evolution of mycelial growth inhibition (%) (mean ± standard error) of *Botrytis cinerea* (**A**), *Penicillium expansum* (**B**), and *Aspergillus niger* (**C**) by different concentrations of *T. kotschyanus* EO (ppm). **A**–**C** mean values not followed by a common letter differ significantly (*** significant at *p* < 0.001).

The antifungal activity of EOs against mycelial growth is shown in Figure 2. Among the fungal strains tested, *B. cinerea* (Figure 3A) was the most susceptible fungus, and it was completely inhibited by all EO treatments. In the cases of *A. niger* (Figure 3B) and *P. expansum* (Figure 3C), mycelial growth was reduced at 250 ppm, being completely inhibited at concentrations of ≥500 ppm (fungicidal effect). In addition, the effects of EOs at 250 ppm were considered fungistatic for *P. expansum* and *A. niger* that were partially inhibited.

Some components of the EO, such as thymol, γ-terpinene, and carvacrol, display strong antifungal activity [53,54,55,56,57]. Particularly for thymol, a monoterpene phenolic compound, previous studies indicated an important effect against the growth of important fruit and food-spoiling fungi, such as *A. niger, Alternaria alternata, B. cinerea, Fusarium oxysporum*, and *Rhizopus oryzae*, that are [58,59,60,61,62]. In a similar way as the results obtained by us, other studies observed that thymol and carvacrol exhibit an important antifungal activity against postharvest pathogens *Botrytis cinerea* [63] and also against spoilage yeasts in wine [64]. Additionally, other research found that carvacrol inhibits the growth of *Penicillium expansum* spores, which agree with our results [65]. The protective effect of *T. kotschyanus* EO can be explained by irreversible damage to fungi membrane and the consequent leakage of the cytoplasmic contents [64], although the effects of other minor components should not be overlooked.

### 2.3. Phytotoxic Activity

**Figure 4:**
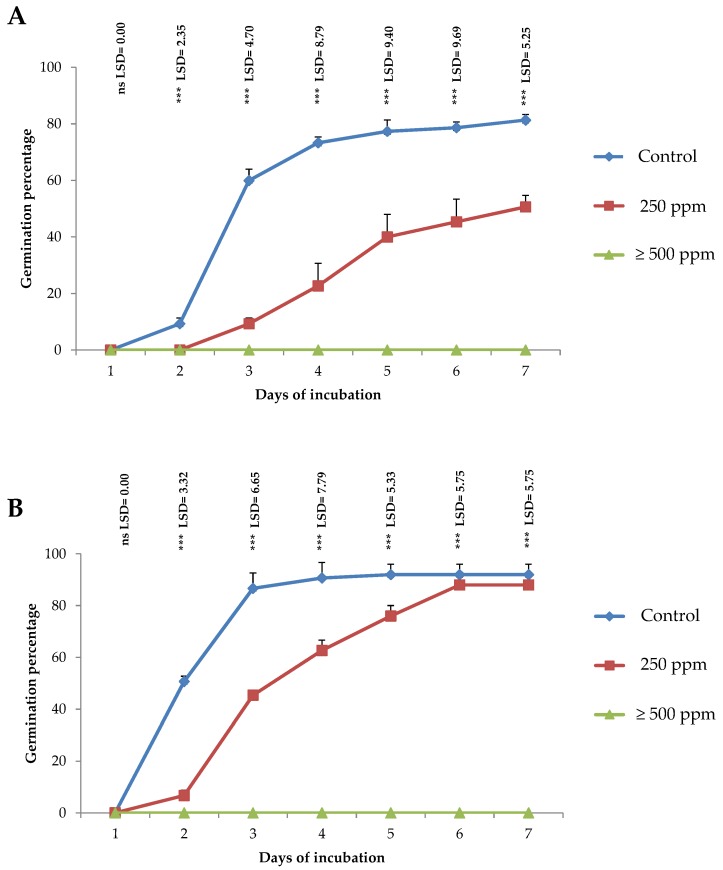
Effect of different concentrations of *T. kotschyanus* EO (μL/L) on the cumulative germination (mean ± standard error) of *Amaranthus retroflexus* (**A**) and *Panicum miliaceum* (**B**). The least significant difference (LSD) indicates the differences between factors.

The effect of EOs on weeds germination indices is shown in Table 2. In both weed species, germination and growth indices were affected by *T. kotschyanus* EO treatments. The final germination percentage (GP) varied significantly (*p* < 0.01) among the different EO concentrations used. For example, seed germination of *A. retroflexus* (Figure 4A) and *P. miliaceum* (Figure 4B) were completely inhibited when the concentrations exceeded 500 ppm. Likewise, the percentage germination of *A. retroflexus* was 81.33% and 0% for control and essential oils more than 500 ppm, respectively. For *P. miliaceum*, such index was 92.00% and 0% for control and EO more than 500 ppm, respectively. Moreover, mean germination time was significantly (*p* < 0.01) influenced by *T. kotschyanus* EO concentration.

The GRI was significantly (*p* < 0.01) influenced by EO concentration, being the highest value found for control (6.50 and 9.65 units for *A. retroflexus* and *P. miliaceum*, respectively), and the lowest was obtained from EO treatments with 500, 750, 1000, and 1500 ppm (0.00 units) for both *A. retroflexus* and *P. miliaceum*. A similar outcome was obtained for root length (RL) and shoot length (ShL). The seeds treated with *T. kotschyanus* EO displayed lower mean values than obtained in the control. The complete inhibition of root and shoot lengths were observed for seeds treated with ≥500 ppm of *T. kotschyanus* EO.

Statistically significant differences (*p* < 0.01) among treatments were also observed in the seedling length (SLL) in both weeds. The highest length was observed in control (3.05 and 8.87 cm), while the lowest values were obtained after the exposition of seeds to more than 500 ppm of EO (0.00 cm) for *A. retroflexus* and *P. miliaceum*, respectively. The EOs decreased (*p* < 0.01) the SLL in a concentration-dependent manner, according to the weeds.

Moreover, it was also found that several concentrations of EOs had a significant effect (*p* < 0.01) on the fresh weight (FW) of seedlings. FW of samples treated with EOs were significantly lower than control for both weeds. Likewise, the vigor index (VI) was affected by EO concentration in both weeds. While the highest VI index was obtained in control (248.33 and 803.67 units for *A. retroflexus* and *P. miliaceum*, respectively), the lowest means values were observed in treatments with more than 500 ppm (0.00 units) of EO. The time to achieve 50% germination (T50 index) varied between the treatments for both studied species. The T50 index increased from 2.57 (control) to 4.17 (250 ppm) days for *A. retroflexus* seeds and from 1.86 (control) to 3.06 (250 ppm) days. Seeds treated with more than 500 ppm of *T. kotschyanus* EO did not germinate. The results obtained for *T. kotschyanus* EO phytotoxic activity are in agreement with other studies in scientific literature. An experiment with four *Thymus daenensis* ecotypes collected in Iran indicated that applying between 400 and 600 μL/L of EO was associated with complete inhibition of *A. retroflexus* seed GP, which consequently reduced the shoot and root fresh weight [66]. In the same line, the EO extracted from *Thymus vulgaris* displayed one of the lowest ED50 (concentration that causes 50% inhibition of seed germination; 0.16 g/L) values to inhibit the germination of *A. retroflexus* seeds among selected essential oils (lemon balm, sage, and tansy, for instance) [67].

The allelopathic effect of *T. kotschyanus* EO on *A. retroflexus* and *P. miliaceum* seeds could be explained by the individual activity of monoterpenes. A study about the phytotoxic effect of pure thymol (10 mg/Petri dish) reported GP of 0.00% and root growth of 0.00% in [68]. Likewise, the treatment with γ-terpinene inhibited the GP (76.5 vs. 32.0 and 21.3% for control and 10 and 20 μL, respectively) and seedling root growth (28.9 vs. 22.0 and 14.5 mm for control and 10 and 20 μL, respectively) of *A. retroflexus* seeds [69].

Moreover, seems reasonable to consider that these two monoterpenes are the main compounds associated with *T. kotschyanus* EO allelopathic activity due to low phytotoxic activity reported for *p*-cymene on *A. retroflexus*, *Chenopodium album*, and *Rumex crispus* seeds [68]. Although the inhibitory mechanism exerted by terpenes on weed seeds remains unclear, previous studies reported relevant effects on cellular proliferation, induction of oxidative stress, and inhibition of DNA synthesis on weed seeds after terpene treatment [70,71].

### 2.4. Insecticidal Activity

The *T. kotschyanus* EO was lethal for both insect species (*O. surinamensis* and *S. oryzae*) used in the experiments. *O. surinamensis* was more susceptible to the effect of EOs than *S. oryzae*, wherein the values of LC_50_ were 4.78 and 13.20 µL/L air, respectively (Table 3).

**Figure 5:**
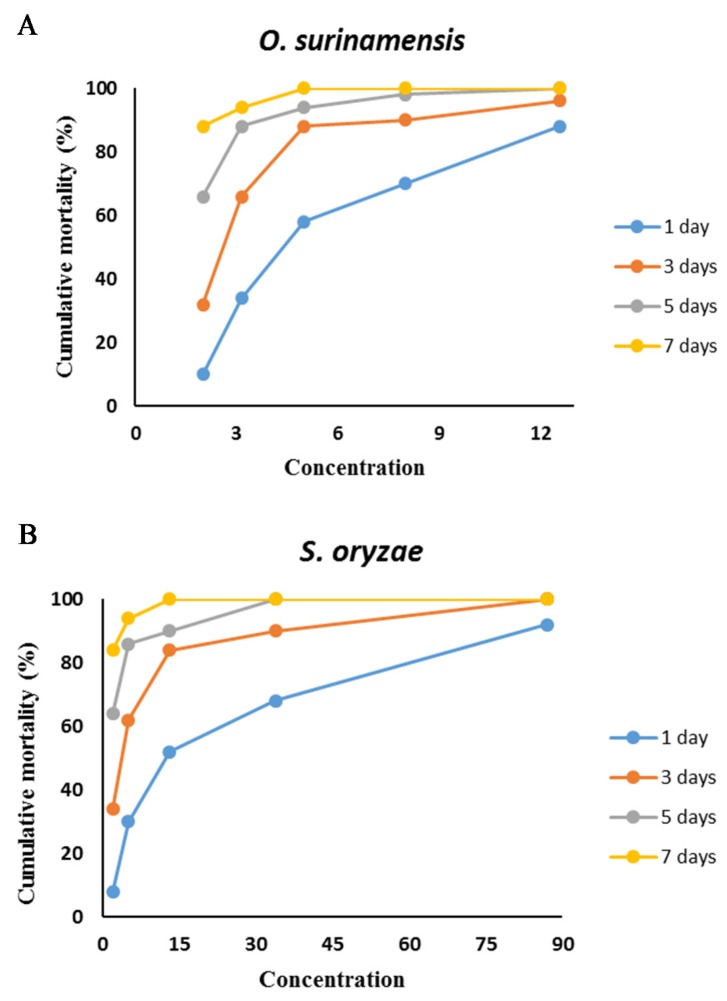
Cumulative mortality of stored pests (**A**: *O. surinamensis* and **B**: *S. oryzae*.exposed) to different concentrations of *T. kotschyanus* essential oil (µL/L air).

**Figure 6:**
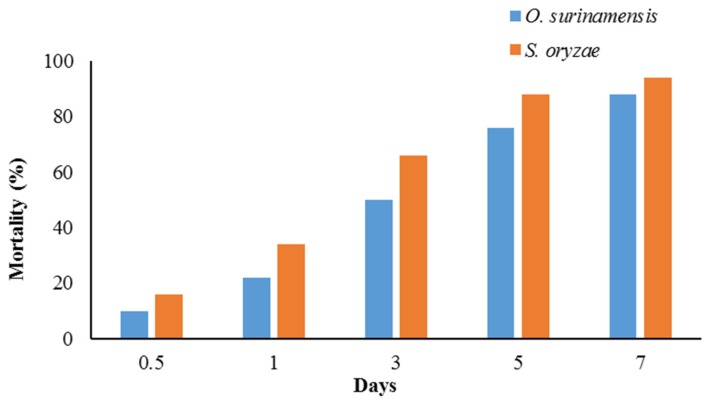
Evolution of pest mortality (%) exposed to 3 µL/L air of *T. kotschyanus* essential oil.

Moreover, LT_50_ values highlighted that the EOs killed *O. surinamensis* faster than *S. oryzae*. Cumulative mortality of *O. surinamensis* (Figure 5A) and *S. oryzae* (Figure 5B) increased daily. Half the population of *O. surinamensis* adults died within 1.57 days, and 95% of insects were killed within 9.17 days after exposure to EO vapors. However, 50 percent and 95 percent of adult rice weevils were killed after 2.36 and 14.60 days of treatment, respectively (Table 4 and Figure 6).

These results are in agreement with data reported by other authors. For instance, the fumigant toxicity effects of commercial thyme EO and selected terpenes, particularly *p*-cymene and thymol, were evaluated against *S. oryzae* [72]. The authors obtained LC_50_ and LC_95_ concentrations of 63.9 and 89.5 μL/L air for thyme EO, respectively. Regarding the individual terpenes, *p*-cymene displayed lower LC_50_ and LC_95_ concentrations (25 and 39 μL/L air, respectively) than those obtained for thymol (69 and 174 μL/L air, respectively). A similar fumigant toxicity effect of thyme EO was reported against *Callosobruchus maculatus* and *Sitophilus granaries* (relevant pests in the storage of legumes and wheat). In this case, the EO extracted from *T. daenensis* Celak displayed LC_50_ concentrations of 4.22 and 6.55 μL/L air for *C. maculatus* and *S. granaries*, respectively. The authors also obtained the LC_90_ concentrations for *C. maculatus* and *S. granaries* (8.21 and 8.73 μL/L air, respectively).

The key role of *T. kotschyanus* EO compounds in the mortality of crop pests is also associated with impairment of important molecular pathways. Particularly for thymol, it was reported that this terpene can influence the GABA-gated chloride channel, which causes hyperexcitation of the central nervous system and can lead to convulsions and death. Another related effect of thymol exposure is modulation of a tyramine receptors cascade that eventually blocks the octopamine receptors and undermines neurological insect functions [73]. Finally, our results illustrated that *T. kotschyanus* has an insecticidal activity against insect pests.

## 3. Materials and Methods

### 3.1. Plant Materials

Aerial parts of *Thymus kotschyanus* plants (voucher number: UHDH-101) in the flowering stage were taken from the Shabestar Region in the East Azerbaijan Province, Iran in 2016. Having a classically semi-arid climate and at the altitude of 1352 m above the sea level (Latitude: 38°19′ N; Longitude: 45°18′ E), this region has a high precipitation rate that often takes place throughout the autumn and winter, whereas there is little rainfall in the summer.

### 3.2. Extraction of Essential Oil

Aerial parts of *T. kotschyanus* were harvested and dried at room temperature, preventing them from taking light. Dried leaves (20 g) were subjected to hydrodistillation through a Clevenger instrument (Urmia University, Urmia, Iran) for 3 h. The resulting EO was poured into screw-capped vials and maintained in darkness at 4 °C for further analysis.

### 3.3. GC and GC-MS Analysis

The analysis of the oil was performed using an Agilent gas chromatograph (GC-FID) (Agilent Technologies, Santa Clara, CA, USA) with a DB-5-fused silica column (30 m × 0.25 mm; 0.25 µm film thickness). Nitrogen was used as the gas carrier at a constant flow of 1.1 mL/min. The oven temperature was programmed from 60 to 250 °C at 5 °C/min and then isothermaled for 10 min. The injector and FID temperatures were set at 250 °C and 280 °C, respectively. The injection volume was 0.1 mL. Samples were injected by splitting, and the split ratio was 1:100. GC-MS analysis was carried out on a Thermoquest Finnigan Trace GC-MS instrument equipped with a DB-5 column (30 m × 0.25 mm; 0.25 µm film thickness) programmed as above, with helium as the carrier gas with a flow rate of 1.1 mL/min and a split ratio of 1:50. The MS operating parameters were: ionization voltage, 70 eV and ion source temperature, 200 °C. Identification of the compounds was performed by comparison of the retention indexes (relative to a homologue C_6_–C_24_ *n*-alkane series) obtained in the same column with those of reference compounds. Additionally, each mass spectra obtained was compared with those from the usual electronic libraries [74,75]. Relative area percentages obtained from GC were used for quantification of the components.

### 3.4. Fungal Isolates

Three post-harvest pathogens fungal isolates, Bot-245 g (*Botrytis cinerea* Pers.), Pen-653mb (*Penicillium expansum* Link), and As-88ma (*Aspergillus niger* Tiegh.), purchased from the fungal culture collection of the Plant Pathology Department, Urmia University (Urmia, Iran) were used in our experiments. The pathogenicity of fungal isolates was previously confirmed (data not shown). Fungal isolates were grown in potato dextrose agar (PDA) medium at 25 ± 2 °C. Only actively growing colonies were used in bioassays [76].

### 3.5. In vitro Antifungal Assays

The antifungal activity of the *T. kotschyanus* essential oil was evaluated through the poison food medium method. Different concentrations of *T. kotschyanus* essential oil (0, 250, 500, 750, 1000, and 1500 µL/L) were prepared in sterile water containing Tween 80 (0.5%, *v*/*v*) and aseptically added to sterile, cooled, molten potato dextrose agar (PDA Merck, Darmstadt, Germany) medium (45 °C). The resulting mixture (EO plus medium) were instantly dispensed onto sterilized glass petri plates (90 mm diameter, 20 mL each) and allowed to solidify under aseptic conditions. A mycelial disk (6-mm-diameter) of the tested fungi, taken from the margins of the actively growing cultures, was placed upside-down at the center of the petri plates.

Inoculated petri plates were incubated in darkness at 25 ± 2 °C. The control was composed of 0.5%Tween 80 in sterile water. Four replicates were used for each treatment, and all the experiments were repeated twice. Antifungal activity of essential oil was measured taking into account the percentage of the mycelial growth inhibition (MGI), calculated by the following the formula [77]:

(1) $$MGI\;\left(\%\right)=\;\frac{\left(dc-dt\right)}{dc}\;\times 100$$

where *dc* was the colony growth diameter in the control and *dt* represented the diameter of colony growth in the treatment.

In the case of no visible growth detected after the incubation with the essential oil, and in order to determine fungistatic and/or fungicidal effects of the essential oil against the tested fungi, the inoculated discs were transferred to new PDA plates and incubated again at 25 ± 2 °C for more than 72 h. If mycelial growth was restarted in PDA medium, the effect was considered fungistatic; otherwise, it was considered fungicidal.

### 3.6. Phytotoxic Activity

Ripe seeds of *Amaranthus retroflexus* L. (redroot pigweed) and *Panicum miliaceum* L. (millet) collected from the North West of Iran (West Azerbaijan Province) on September, 2016 were used to investigate the phytotoxic effect exhibited by the EO. Seeds were stored in paper bags for a span of four weeks at room temperature. The viability of the seeds and their germinability were checked prior to the experiments. Surfaces of seeds were sterilized through a two-step procedure (rinse for 30 s with 70% ethyl-alcohol and a subsequent treatment with a 10% sodium hypochlorite solution for 20 min), then washed three times with sterile distilled water, and finally, air-dried in aseptic conditions under a laminar hood. Fifty seeds from each weed were placed in Petri dishes containing two layers of filter-paper (Whatman No. 2). To make exact concentrations of EO in water (0, 250, 500, 750, 1000, and 1500 µL/L), first a stock of EO in dimethyl sulfoxide (DMSO)/water (1%, *v*/*v*) was prepared. Ultimately, 10 mL of each concentration was poured into the petri dishes. In the controls, 1% DMSO in water was used. Each treatment had five replicates, and all the experiments were replicated twice. The petri dishes containing seeds were sealed by plastic paraffin film tape. Then, petri dishes were kept in a germinator set at 25 °C with a 16-h photoperiod of 28–36 mM/m^2^ s.

In this experiment, germination percentage (GP); mean germination time (MGT); germination rate index (GRI); vigor index (VI); root, shoot, and seedling lengths (RL, ShL, and SLL, respectively); and T50 index were measured. The GP was expressed as the ratio of germinated seed to the total of the seeds. The MGT and GRI were calculated using the following formula [78]:

(2) $$\mathrm{MGT}=\frac{\sum n.d}{N}$$

where *n* is the number of seeds germinated on each day, *d* is the days from the beginning of the germination test, and *N* represents the final germinated seeds. The GRI:

(3) $$\mathrm{GRI}=\sum \frac{\left(\mathrm{number}\;\mathrm{of}\;\mathrm{germinated}\;\mathrm{seeds}\;\mathrm{since}\;n-1\right)}{n}$$

where *n* represents the days of incubation. At the end of the incubation, root, shoot, and seedling lengths were also measured, and the seed vigor index (VI) was obtained using the equation [79]:

(4) VI=[SLL (cm)x GP]

The T50 value was calculated in terms of days needed for germination of 50% of the seeds.

### 3.7. Insecticidal Activity

The fumigant toxicity of *T. kotschyanus* EO was assessed using two model insect species, which are mainly the infesting of food products during their storage, namely the saw-toothed grain beetle (*Oryzaephilus surinamensis*) and the so-called rice weevil (*Sitophilus oryzae*). LC_50_ (median lethal concentration) and LT_50_ (median lethal time) values were used as parameters to calculate the insecticidal strength of the EO. After preliminary bioassays, 6-cm-diameter disks of filter papers (Whatman No. 1) were impregnated with different concentrations of the essential oil (2–87 µL/L air for *S. oryzae* and 2–12.6 µL/L air for *O. surinamensis*) without any solvent. The disks were mounted on the underside of tightly screwed caps of 250 mL glass vials. Ten newly emerged adults of each insect were introduced into each vial. Combinations of different concentrations and exposure times (1–7 days) were replicated five times. In the controls, only filter papers were used. Vials were kept in darkness, 70% ± 5% RH and 28 ± 1 °C. Mortality percentage was recorded at 24-h intervals until 7 days. Insects with no reaction after physical stimulation (leg or antennal movements) were considered as dead.

### 3.8. Statistical Analysis

Statistical analyses of the data (antifungal and phytotoxic activity) were performed using MSTAT-C statistical software (Michigan State University, East Lansing, MI, USA), and means were separated by DMRT at 0.01 probability level. In the case of insecticidal activity, all the experiments were replicated five times. Data were analyzed using SPSS V22.0 software (IBM, Armonk, NY, USA).

## 4. Conclusions

The analysis of *T. kotschyanus* EO (yield around 3.5%) composition collected in the Shabestar Region (Iran) revealed that thymol is the main compound, followed by γ-terpinene and *p*-cymene. Moreover, our results also showed that *T. kotschyanus* EOs can be considered as an efficient natural compound to control post-harvest fungal diseases (*A. niger* and *P. expansum*; fungicidal effect at concentration ≥500 ppm); weeds (*A. retroflexus* and *P. miliaceum*; ≥500 ppm); and harmful insects (*O. surinamensis* and *S. oryzae*). Therefore, taking into account the several issues related to the harmful effects on the environment and on the health associated with synthetic pesticides, and the remarkable fungicidal properties of *T. kotschyanus* active components, the EO evaluated in the present study can be certainly considered as a good alternative in the post-harvest pest management.

## Figures and Tables

**Table 1:** Percentage chemical composition and retention indices of the essential oil of *Thymus kotschyanus*.

No	Compounds	Tn	RI	RI-L	Percentage
1	α-Thujene	3.98	926	925	1.38 ± 0.01
2	α-Pinene	4.11	934	932	0.96 ± 0.00
3	Camphene	4.37	949	952	0.62 ± 0.00
4	β-Pinene	4.86	978	978	0.53 ± 0.00
5	3-Octanone	4.97	984	984	0.16 ± 0.00
6	β-Myrcene	5.07	990	991	1.36 ± 0.01
7	3-Octanol	5.16	995	994	0.15 ± 0.00
8	α-Phellandrene	5.37	1006	1006	0.27 ± 0.00
9	3-Carene	5.49	1011	1011	0.08 ± 0.00
10	α-Terpinene	5.62	1017	1017	2.1 ± 0.01
11	*p*-Cymene	5.82	1026	1026	5.56 ± 0.02
12	Limonene	5.89	1030	1030	0.52 ± 0.00
13	1,8-Cineol	5.95	1032	1033	2.82 ± 0.02
14	γ-Terpinene	6.56	1060	1060	6.67 ± 0.03
15	cis-Sabinene hydroxide	6.76	1069	1070	1.66 ± 0.01
16	α- Terpinolen	7.21	1090	1090	0.12 ± 0.00
17	trans-Sabinene hydrate	7.48	1102	1104	0.57 ± 0.00
18	Camphor	8.6	1148	1148	0.36 ± 0.00
19	Borneol	9.18	1171	1173	1.82 ± 0.01
20	Terpinen-4-ol	9.44	1182	1182	0.88 ± 0.00
21	α-Terpineol	9.79	1196	1195	0.17 ± 0.00
22	cis-α-terpineol	10.02	1205	1209	0.13 ± 0.00
23	Carvacrol methyl ether	11.12	1247	1246	2.94 ± 0.01
24	Thymol	12.84	1313	1311	60.48 ± 0.78
25	Carvacrol	12.94	1317	1316	3.02 ± 0.01
26	Thymol acetate	13.97	1358	1357	0.13 ± 0.00
27	E-Caryophyllene	15.62	1424	1422	2.18 ± 0.01
28	Aromandendrene	16.08	1443	1440	0.09 ± 0.00
29	α-Humulene	16.44	1458	1457	0.08 ± 0.00
30	γ-Muurolene	16.99	1480	1479	0.09 ± 0.00
31	Virdiflorene	17.45	1499	1497	0.13 ± 0.00
32	β-Bisabolene	17.74	1512	1511	1.36 ± 0.01
33	δ-Cadinene	18.1	1527	1526	0.16 ± 0.00
34	(E)-α-Bisabolene	18.53	1545	1545	0.17 ± 0.00
35	Spathulenol	19.42	1583	1582	0.08 ± 0.00
36	Caryophyllene oxide	19.53	1588	1587	0.21 ± 0.00

**Table 2:** Influence of various concentrations of *T. kotschyanus* essential oil in seed germination indices of *A. retroflexus* and *P. miliaceum* at the end of incubation time. GP: germination percentage, MGT: mean germination time, GRI: germination rate index, RL: root length, ShL: shoot length, SLL: seedling length, FW: fresh weight, VI: vigor index, and T50: the time to reach 50% germination.

Treatments	GP (%)	MGT (day)	GRI	RL (cm)	ShL (cm)	SLL (cm)	FW (mg)	VI	T50
***Amaranthus retroflexus***									
Control	81.33 ± 2.0	3.22 ± 0.31	6.50 ± 0.13	1.54 ± 0.12	1.52 ± 0.00	3.05 ± 0.13	0.02 ± 0.00	248.33 ± 16.81	2.57 ± 0.01
250 ppm	50.67 ± 4.0	4.70 ± 0.28	2.86 ± 0.35	0.45 ± 0.12	0.00	0.46 ± 0.13	0.01 ± 0.00	23.52 ± 8.44	4.17 ± 0.29
500 ppm	0.00	0.00	0.00	0.00	0.00	0.00	0.00	0.00	0.00
750 ppm	0.00	0.00	0.00	0.00	0.00	0.00	0.00	0.00	0.00
1000 ppm	0.00	0.00	0.00	0.00	0.00	0.00	0.00	0.00	0.00
1500 ppm	0.00	0.00	0.00	0.00	0.00	0.00	0.00	0.00	0.00
LSD	5.25	0.45	0.43	0.19	0.016	0.20	0.01	19.3	0.33
Prob	***	***	***	***	***	***	***	***	***
***Panucum miliaceum***									
Control	92 ± 4.00	2.52 ± 0.08	9.65 ± 0.28	5.54 ± 1.09	3.23 ± 0.27	8.78 ± 1.36	0.12 ± 0.03	803.67 ± 91.0	1.86 ± 0.03
250 ppm	88 ± 4.00	3.83 ± 0.15	6.30 ± 0.11	1.06 ± 0.16	0.95 ± 0.10	2.01 ± 0.12	0.02 ± 0.00	176.44 ± 2.1	3.06 ± 0.05
500 ppm	0.00	0.00	0.00	0.00	0.00	0.00	0.00	0.00	0.00
750 ppm	0.00	0.00	0.00	0.00	0.00	0.00	0.00	0.00	0.00
1000 ppm	0.00	0.00	0.00	0.00	0.00	0.00	0.00	0.00	0.00
1500 ppm	0.00	0.00	0.00	0.00	0.00	0.00	0.00	0.00	0.00
LSD	5.75	0.17	0.34	1.23	0.30	1.48	0.02	106.7	0.06
Prob	***	***	***	***	***	***	***	***	***

Data represent means of three replicates compared by Duncan´s multiple range test (DMRT) at *p* < 0.01. *** Significant at (*p* < 0.001).

**Table 3:** Lethal concentration values of *T. kotschyanus* essential oil on *O. surinamensis* and *S. oryzae*.

Insect	LC_50_ (µL·L^−1^)	LC_95_ (µL·L^−1^)	Slope ± SE	Intercept ± SE	*χ*^2^ (df = 3)	*p*-Value
*O. surinamensis*	4.78	17.98	2.86 ± 0.34	−1.94 ± 0.26	2.04	0.57
	(4.12–5.51)	(13.63–27.63)				
*S. oryzae*	13.2	150.22	1.56 ± 0.18	−1.75 ± 0.22	1.83	0.61
	(10.08–17.29)	(90.73–320.93)				

**Table 4:** Lethal time values of *T. kotschyanus* essential oil on *O. surinamensis* and *S. oryzae.*

Insect	LT_50_ (days)	LT_95_ (days)	Slope ± SE	Intercept ± SE	*χ*^2^ (df = 3)	*p*-Value
*O. surinamensis*	1.57	9.17	2.14 ± 0.23	−0.42 ± 0.12	1.73	0.63
	(1.26–1.91)	(6.64–14.62)				
*S. oryzae*	2.36	14.6	2.08 ± 0.23	−0.78 ± 0.13	2.47	0.48
	(1.93–2.89)	(10.12–25.22)				
